# Evolution of guideline-based male infertility responses by large language models between 2025 and 2026: a comparative analysis of ChatGPT, Perplexity, and DeepSeek

**DOI:** 10.1186/s12610-026-00327-z

**Published:** 2026-10-16

**Authors:** Burak Elmaağaç, Ali Yasin Özercan, Murat Demir, Murat Gül

**Affiliations:** 1 Department of Urology, University of Health Sciences, Kayseri Faculty of Medicine, Kayseri City Hospital, Kayseri, Türkiye; 2 Department of Urology, Kayseri City Hospital, Kayseri, Türkiye; 3 Department of Urology, Van Yüzüncü Yıl University, Faculty of Medicine, Van, Türkiye; 4 Department of Urology, Selçuk University, Faculty of Medicine, Konya, Türkiye

**Keywords:** Large language models, Male infertility, Artificial intelligence, ChatGPT, Perplexity, DeepSeek, EAU guidelines, Health information quality, Patient safety, Readability, grands modèles de langage, infertilité masculine, intelligence artificielle, ChatGPT, Perplexity, DeepSeek, recommandations de l’EAU, qualité de l’information en santé, sécurité des patients, lisibilité

## Abstract

**Contexte:**

Les grands modèles de langage (LLM) sont de plus en plus utilisés par les patients et les cliniciens pour obtenir des informations médicales, mais leurs performances peuvent évoluer au fil du temps à mesure que les modèles sont mis à jour. L’infertilité masculine est un domaine étroitement encadré par des recommandations, dans lequel des informations inexactes ou incomplètes peuvent influer sur l’évaluation diagnostique, le conseil aux patients et les attentes thérapeutiques. Cette étude a évalué l’évolution, entre 2025 et 2026, des performances de ChatGPT, Perplexity et DeepSeek dans leurs réponses à des questions sur l’infertilité masculine élaborées à partir de recommandations cliniques.

**Résultats:**

Au total, 528 réponses de chatbots ont été évaluées à partir de 88 questions sur l’infertilité masculine issues des recommandations de l’Association européenne d’urologie (EAU), auxquelles ChatGPT, Perplexity et DeepSeek ont répondu en 2025 et en 2026. En 2025, des différences significatives entre les modèles ont été observées dans les quatre domaines évalués: Perplexity a obtenu les meilleurs scores pour la qualité du contenu et l’évaluation par les experts, tandis que DeepSeek affichait les performances les plus faibles. En 2026, les scores DISCERN ont convergé entre les modèles, alors que des différences significatives persistaient en matière de lisibilité, de score global de qualité et d’avis des experts. ChatGPT s’est significativement amélioré dans les quatre domaines évalués et a obtenu le meilleur score global de qualité en 2026. DeepSeek a enregistré l’amélioration la plus marquée, notamment pour les scores DISCERN et d’avis des experts. La lisibilité des réponses de Perplexity s’est améliorée, mais cette évolution ne s’est pas accompagnée d’une amélioration parallèle du score global de qualité ni de l’utilité clinique évaluée par les experts. Des risques potentiels pour la sécurité des patients ont été relevés dans sept des 88 réponses fournies par DeepSeek en 2025, tandis qu’aucun risque de ce type n’a été constaté dans les réponses de 2026. Aucune des 528 réponses ne contenait de recommandation explicite considérée comme susceptible de causer un préjudice immédiat ou grave aux patients.

**Conclusions:**

Les performances ont évolué selon des modalités propres à chaque modèle, plutôt que de manière uniformément progressive. Les résultats correspondent à un échantillon de réponses obtenues lors d’une première interrogation à des questions clairement formulées et dérivées de recommandations, plutôt qu’à des estimations robustes des performances ou de la reproductibilité des modèles. Les grands modèles de langage (LLM) peuvent faciliter l’accès aux informations sur l’infertilité masculine, mais une vérification auprès de sources faisant autorité et une supervision humaine appropriée demeurent nécessaires.

**Supplementary Information:**

The online version contains supplementary material available at https://doi.org/10.1186/s12610-026-00327-z.

## Abstract (fr)

**Background:**

Large language models (LLMs) are increasingly used by patients and clinicians to obtain medical information, yet their performance may change over time as models are updated. Male infertility is a guideline-sensitive field in which inaccurate or incomplete information may affect diagnostic evaluation, counseling, and treatment expectations. This study evaluated the year-on-year evolution of ChatGPT, Perplexity, and DeepSeek in responding to guideline-derived male infertility questions between 2025 and 2026.

**Results:**

A total of 528 chatbot responses were evaluated from 88 European Association of Urology (EAU) guideline-derived male infertility questions answered by ChatGPT, Perplexity, and DeepSeek in 2025 and 2026. In 2025, significant inter-model differences were observed across all four scored domains, with Perplexity achieving the highest content-quality and expert-rated scores and DeepSeek showing the weakest performance. In 2026, DISCERN scores converged across models, whereas significant differences persisted for readability, Global Quality Score, and expert opinion. ChatGPT showed significant improvement across all four scored domains and achieved the highest Global Quality Score in 2026. DeepSeek demonstrated the most pronounced improvement, particularly in DISCERN and expert opinion scores. Perplexity improved in readability but did not show a parallel positive change in Global Quality Score or expert-rated clinical usefulness. Potential patient-safety concerns were identified in seven of the 88 DeepSeek responses from 2025, whereas no such concern was identified in the 2026 responses. None of the 528 responses contained an explicit recommendation considered likely to cause immediate or serious patient harm.

**Conclusions:**

Performance changed in a model-specific rather than uniformly progressive manner. The findings represent sampled first-pass responses to clearly formulated guideline-derived questions rather than stable estimates of model performance or reproducibility. LLMs may support access to male infertility information, but verification against authoritative sources and appropriate human oversight remain necessary.

## Background

Infertility is a major reproductive health issue, and male factors contribute substantially to the diagnostic work-up of infertile couples [1]. The evaluation of male infertility requires careful interpretation of semen analysis, hormonal findings, testicular assessment, imaging results, genetic indications, and treatment options. Therefore, accurate and guideline-based counseling is essential to avoid incomplete evaluation, inappropriate reassurance, unnecessary testing, or misleading treatment expectations [2, 3].

Large language models (LLMs) are increasingly used by both patients and clinicians to obtain medical information. Publicly accessible platforms such as ChatGPT, Perplexity, and DeepSeek can generate rapid, fluent, and apparently authoritative answers to clinical questions [4–6]. However, fluency does not necessarily indicate accuracy, completeness, source reliability, or consistency with guideline-based care [4, 5]. This is particularly relevant in male infertility, where recommendations often depend on clinical context, reproductive goals, female partner factors, and current evidence [2, 3].

A further challenge is that LLM performance is not static. Chatbot outputs may change over time because of model updates, altered retrieval mechanisms, changes in source access, and modifications in response-generation behavior. In contrast, the male infertility recommendations relevant to the present question set remained largely stable between the 2025 and 2026 European Association of Urology Sexual and Reproductive Health Guidelines [2, 3]. This provided a methodological opportunity to reassess the same guideline-derived clinical questions one year later and evaluate temporal changes in chatbot performance against a largely unchanged clinical reference standard.

The present study evaluated the year-on-year evolution of ChatGPT, Perplexity, and DeepSeek in responding to 88 male infertility-related clinical questions derived from EAU guideline recommendations. We aimed to determine whether each model’s performance improved, deteriorated, or remained stable between 2025 and 2026 in terms of reliability, overall information quality, readability, and expert-rated clinical usefulness.

## Materials and methods

### Study design and reporting framework

This study was designed as a repeated cross-sectional comparative evaluation of large language model-generated responses to male infertility-related clinical questions. The same guideline-derived question set was submitted to three publicly accessible large language models in 2025 and again in 2026. This design was chosen to assess the temporal evolution of chatbot performance over one year while keeping the clinical reference standard largely unchanged.

The study was reported in accordance with the TRIPOD-LLM reporting guideline, where applicable [7]. Because the study did not involve patient-level data, electronic health records, clinical outcome prediction, or human participants, several checklist items were not applicable. The study design, model selection, guideline source, prompt development process, model interrogation protocol, primary evaluation criteria, expert review process, and statistical analysis were predefined. The evaluated LLMs were used solely as the objects of assessment and did not contribute to study design, data interpretation, authorship, or final manuscript approval.

### Guideline source and rationale for repeated assessment

The clinical question set was originally developed from the 2025 European Association of Urology Sexual and Reproductive Health Guidelines, focusing specifically on male infertility recommendations [2]. Before the second assessment period, the 2026 EAU Sexual and Reproductive Health Guidelines were reviewed. Because the 2026 document represented a limited update and the male infertility recommendations relevant to the original question set remained largely stable, the same 88 clinical questions were retained for repeated assessment [3].

This approach allowed the study to focus on changes in model performance rather than changes in the underlying guideline content. Therefore, the purpose of the repeated assessment was not to compare the 2025 and 2026 EAU guideline documents, but to determine whether chatbot-generated responses to the same guideline-based clinical questions improved, deteriorated, or remained stable over time.

### AI model selection

Three widely accessible large language models were evaluated: ChatGPT, Perplexity, and DeepSeek. These platforms were selected because they are publicly available, commonly used for medical information seeking, and represent different response-generation frameworks. ChatGPT was included as a general-purpose conversational large language model. Perplexity was selected because of its retrieval-supported and citation-enabled response structure. DeepSeek was included as a rapidly expanding publicly accessible model with increasing visibility in general and medical information-seeking contexts. The comparator set was intentionally limited to freely accessible, general-purpose, consumer-facing platforms because the study aimed to evaluate the tools most readily available to patients and clinicians seeking medical information in real-world settings. Medical-specific or clinically grounded retrieval-augmented systems were considered outside the predefined scope because they represent a different category of tools in terms of intended use, access, and degree of domain-specific knowledge integration.

The first assessment was conducted in May 2025 using ChatGPT-4o, Perplexity AI, and DeepSeek R1. The repeated assessment was conducted in May 2026 using ChatGPT-5.5, Perplexity Sonar, and DeepSeek V4. All platforms were accessed through their freely available standard user-facing web interfaces. No paid subscription or API-based access was used. The platform name, visible model/version information, assessment month and year, and access mode were recorded to improve transparency and reproducibility.

### Development of clinical questions

A total of 88 English clinical questions were formulated through direct transformation of recommendation statements and clinically relevant information from the EAU male infertility guideline section [2]. The aim was to convert guideline-based content into concise, neutral, information-seeking prompts while preserving the intended meaning of the original recommendations.

Each prompt was recorded together with its corresponding source statement in a master spreadsheet before model interrogation. This ensured transparency, traceability, and reproducibility of the question development process. The same 88 prompts were used for all three models and for both assessment years. Representative examples of this transformation process are shown in Table 1.

**Table 1:** Examples of guideline-derived statements and corresponding AI prompts

Guideline-derived statement	Corresponding AI prompt
Infertility affects approximately 15% of couples of reproductive age.	What percentage of couples of reproductive age are infertile?
Infertility evaluation should be performed in couples who fail to conceive within 12 months.	When should infertility evaluation be performed?
A male factor can be identified in approximately 50% of infertile couples.	In what percentage of infertile couples can a male factor be identified?
Testicular volume may be assessed using a Prader orchidometer or testicular ultrasound.	How should testicular volume be assessed?

All prompts were written in formal English using neutral wording. No leading phrasing, contextual cues, multipart prompts, sequential questioning, or follow-up prompts were used. This standardized format was selected to allow direct comparison of first-pass responses across models and between assessment years.

### Model interrogation protocol

In the May 2025 assessment, each of the 88 prompts was submitted separately to ChatGPT, Perplexity, and DeepSeek. The same procedure was repeated in May 2026 using the identical prompt set. For both assessment years, each prompt was submitted in a new private chat session with memory disabled and no previous conversation history. Each prompt was therefore treated as a standalone query to minimize memory effects and cross-question contamination.

Only the first complete response generated by each model was retained for analysis. Responses were captured exactly as returned by the platforms, without editing, summarization, correction, translation, or post-processing. No follow-up questions, clarifying prompts, regeneration attempts, or iterative refinements were performed. This protocol was selected to reflect a real-world first-query scenario, which is a common pattern of use among patients and clinicians seeking rapid medical information.

### Evaluation criteria and expert review

All AI-generated responses were evaluated independently by two academic urologists with expertise in male infertility. Scoring was performed in a blinded fashion with respect to model identity and assessment year. Four scored evaluation domains were used: reliability, overall information quality, readability, and expert-rated clinical usefulness.

Reliability was assessed using the modified short-form DISCERN instrument [8]. This tool evaluates the reliability and transparency of health information using five binary items, with each item scored as 1 for “yes” and 0 for “no,” resulting in a total score ranging from 0 to 5. The evaluated items included clarity of aims, use of reliable information sources, balance and lack of bias, inclusion of additional sources for patient reference, and acknowledgment of uncertainty or gaps in knowledge.

Overall information quality and educational usefulness were assessed using the Global Quality Score (GQS) [9]. GQS is a five-point Likert-type scale ranging from 1, indicating poor quality, to 5, indicating excellent quality.

Readability was assessed using the Gunning Fog Index (GFI), which estimates the years of formal education required to understand a text on first reading [10, 11]. Higher GFI values indicate greater linguistic complexity and lower readability for general audiences. In this study, GFI was calculated using the same publicly available online calculator for both assessment years [10].

In addition to these structured instruments, each response underwent expert review using a five-point scale ranging from 1, indicating very poor clinical usefulness, to 5, indicating excellent clinical usefulness. This domain specifically considered guideline adherence, clinical accuracy, completeness, and practical relevance in the context of male infertility counseling.

Separately, all responses were evaluated for misleading, unsafe, or potentially harmful content. A response was considered to raise a potential patient-safety concern if it contained inaccurate, incomplete, or insufficiently qualified information that could plausibly lead to inappropriate clinical interpretation, testing, treatment, or delayed evaluation if acted upon without clinician verification.

### Inter-rater agreement

All responses were independently evaluated by two academic urologists with expertise in male infertility (B.E. and M.D.). Final scores were calculated by averaging the two reviewers’ scores for each response. Inter-rater agreement was assessed using the intraclass correlation coefficient, which was 0.87, indicating strong agreement.

### Statistical analysis

Statistical analyses were performed using IBM SPSS Statistics version 22.0 (IBM Corp., Armonk, NY, USA). Since the data were not normally distributed, results were presented as median and interquartile range (IQR). Inter-model comparisons for each assessment year were performed using the Kruskal–Wallis test, followed by Dunn’s post hoc test with Bonferroni correction when appropriate. Within-model comparisons between 2025 and 2026 were performed using the Wilcoxon signed-rank test. A two-sided *p* value < 0.05 was considered statistically significant. Safety findings were summarized descriptively using counts and percentages.

## Results

A total of 528 chatbot responses were evaluated, corresponding to 88 guideline-derived male infertility questions answered by three models in two assessment years. Inter-model comparisons for each year are summarized in Table 2.

**Table 2:** Inter-model comparison of AI-Generated health information quality scores by assessment year

Year	Outcome measure	ChatGPT Median [IQR]	Perplexity Median [IQR]	DeepSeek Median [IQR]	p-value*
**2025**	**Gunning Fog Index**	15.68 [14.19–17.23]	17.39 [14.94–18.84]	15.30 [14.32–16.84]	**<0.001**
**2025**	**DISCERN**	4 [4–4.25]	5 [5–5]	3 [3–4]	**<0.001**
**2025**	**GQS**	4 [4–5]	5 [4–5]	4 [3–4]	**<0.001**
**2025**	**Expert Opinion**	4 [4–5]	5 [4–5]	3 [3–4]	**<0.001**
**2026**	**Gunning Fog Index**	14.99 [13.26–16.32]	16 [13.75–17.46]	14.3 [13.3–15.8]	**<0.001**
**2026**	**DISCERN**	5 [4–5]	5 [4–5]	5 [4–5]	0.106
**2026**	**GQS**	5 [4–5]	4 [4–5]	4 [4–5]	**0.012**
**2026**	**Expert Opinion**	5 [4–5]	4 [4–4.25]	5 [4–5]	**<0.001**

^*^Kruskal-Wallis test. Bold *p*-values indicate statistical significance (*p* < 0.05). Values are expressed as Median [IQR: Q1–Q3]. GFI = Gunning Fog Index; GQS = Global Quality Score. *n* = 88 per model per assessment year

In the 2025 assessment, statistically significant differences were observed among ChatGPT, Perplexity, and DeepSeek across all four scored domains. For readability, Perplexity had the highest Gunning Fog Index score, indicating greater linguistic complexity, whereas ChatGPT and DeepSeek showed lower and comparable scores. Perplexity also achieved the highest median DISCERN score, GQS, and expert opinion score, while DeepSeek showed the lowest median values for DISCERN and expert opinion. Overall inter-model differences in 2025 were statistically significant for the Gunning Fog Index, DISCERN, GQS, and expert-opinion scores.

In the 2026 reassessment, significant inter-model differences persisted for Gunning Fog Index, GQS, and expert opinion scores. However, DISCERN scores no longer differed significantly among the three models. All models showed a median DISCERN score of 5, with overlapping interquartile ranges. For GQS, ChatGPT achieved the highest median score, whereas Perplexity and DeepSeek had lower median scores. For expert opinion, ChatGPT and DeepSeek achieved higher median scores than Perplexity. Perplexity continued to show the highest Gunning Fog Index score, indicating that its responses remained more linguistically complex than those of the other models.

Post hoc pairwise comparisons are presented in Table 3. In 2025, Perplexity differed significantly from both ChatGPT and DeepSeek for DISCERN, GQS, and expert opinion scores. ChatGPT also differed significantly from DeepSeek in these domains. For Gunning Fog Index, Perplexity had significantly higher scores than both ChatGPT and DeepSeek, whereas no significant difference was observed between ChatGPT and DeepSeek.

**Table 3:** Post-hoc pairwise comparisons (Dunn test with Bonferroni correction)

Outcome measure	Comparison	2025		2026	
		Adjusted p-value	Significance	Adjusted p-value	Significance
**Gunning Fog Index**	ChatGPT vs. Perplexity	**0.002**	**	0.061	ns
**Gunning Fog Index**	ChatGPT vs. DeepSeek	1.000	ns	0.571	ns
**Gunning Fog Index**	Perplexity vs. DeepSeek	**<0.001**	***	**<0.001**	***
**DISCERN**	ChatGPT vs. Perplexity	**<0.001**	***	N/A	—
**DISCERN**	ChatGPT vs. DeepSeek	**<0.001**	***	N/A	—
**DISCERN**	Perplexity vs. DeepSeek	**<0.001**	***	N/A	—
**GQS**	ChatGPT vs. Perplexity	**<0.001**	***	**0.024**	*
**GQS**	ChatGPT vs. DeepSeek	**<0.001**	***	**0.037**	*
**GQS**	Perplexity vs. DeepSeek	**<0.001**	***	1.000	ns
**Expert Opinion**	ChatGPT vs. Perplexity	**0.002**	**	**<0.001**	***
**Expert Opinion**	ChatGPT vs. DeepSeek	**<0.001**	***	0.253	ns
**Expert Opinion**	Perplexity vs. DeepSeek	**<0.001**	***	**<0.001**	***

Dunn test with Bonferroni correction for multiple comparisons. Highlighted cells indicate statistically significant pairwise differences (*p* < 0.05). N/A = post-hoc not applicable (Kruskal-Wallis *p* ≥ 0.05 for that metric/year). *** *p* < 0.001; ** *p* < 0.01; * *p* < 0.05; ns = not significant

In 2026, pairwise comparisons showed a different pattern. For Gunning Fog Index, Perplexity remained significantly different from DeepSeek, while the difference between ChatGPT and Perplexity was no longer statistically significant. For DISCERN, post hoc testing was not applicable because the overall inter-model difference was not statistically significant. For GQS, ChatGPT scored significantly higher than both Perplexity and DeepSeek, whereas Perplexity and DeepSeek did not differ significantly. For expert opinion, Perplexity scored significantly lower than both ChatGPT and DeepSeek, while no significant difference was observed between ChatGPT and DeepSeek.

Within-model comparisons between 2025 and 2026 are shown in Table 4. ChatGPT demonstrated significant improvement across all four scored domains. Its Gunning Fog Index decreased from 15.68 to 14.99, indicating improved readability, while DISCERN, GQS, and expert opinion scores each increased by one median point.

**Table 4:** Within-model comparison of quality scores between assessment Years (2025 vs. 2026)

Model	Outcome measure	2025 Median [IQR]	2026 Median [IQR]	Δ Median (2026–2025)	p-value†
**ChatGPT**	**Gunning Fog Index**	15.68 [14.19–17.23]	14.99 [13.26–16.32]	−0.69	**<0.001**
**ChatGPT**	**DISCERN**	4 [4–4.25]	5 [4–5]	+1.00	**<0.001**
**ChatGPT**	**GQS**	4 [4–5]	5 [4–5]	+1.00	**<0.001**
**ChatGPT**	**Expert Opinion**	4 [4–5]	5 [4–5]	+1.00	**<0.001**
**Perplexity**	**Gunning Fog Index**	17.39 [14.94–18.84]	16 [13.75–17.46]	−1.39	**<0.001**
**Perplexity**	**DISCERN**	5 [5–5]	5 [4–5]	+0.00	**<0.001**
**Perplexity**	**GQS**	5 [4–5]	4 [4–5]	−1.00	**<0.001**
**Perplexity**	**Expert Opinion**	5 [4–5]	4 [4–4.25]	−1.00	**<0.001**
**DeepSeek**	**Gunning Fog Index**	15.3 [14.32–16.84]	14.3 [13.3–15.8]	−1.00	**<0.001**
**DeepSeek**	**DISCERN**	3 [3–4]	5 [4–5]	+2.00	**<0.001**
**DeepSeek**	**GQS**	4 [3–4]	4 [4–5]	+0.00	**<0.001**
**DeepSeek**	**Expert Opinion**	3 [3–4]	5 [4–5]	+2.00	**<0.001**

^†^ Wilcoxon signed-rank test (paired, 2025 vs. 2026 within each model). Bold *p*-values indicate statistical significance (*p* < 0.05). Values are expressed as Median [IQR: Q1–Q3]. Δ Median = difference in medians (2026 minus 2025). *n* = 88 per model

Perplexity showed a significant reduction in Gunning Fog Index from 17.39 to 16.00, indicating improved readability. However, its content-related scores showed a mixed pattern. The median DISCERN score remained 5, but the interquartile range widened in 2026. In contrast, both GQS and expert opinion scores decreased by one median point.

DeepSeek showed the most pronounced improvement over time. Its Gunning Fog Index decreased from 15.30 to 14.30. DISCERN increased from a median of 3 to 5, and expert opinion increased from a median of 3 to 5. GQS remained at a median of 4, although the distribution shifted upward in 2026. All within-model comparisons between 2025 and 2026 were statistically significant. The safety evaluation identified potential patient-safety concerns in seven of the 88 DeepSeek responses from 2025 (8.0%). These responses contained inaccurate, incomplete, or insufficiently qualified information and had also received low GQS and expert-opinion scores, consistent with their limited clinical quality and usefulness. No potential patient-safety concern was identified in the 2026 responses. None of the 528 responses contained an explicit diagnostic or therapeutic recommendation considered likely to cause immediate or serious patient harm.

Overall, the 2025 assessment showed clear inter-model differences, with Perplexity achieving the highest content-quality and expert-rated scores and DeepSeek showing the weakest performance. By 2026, DISCERN scores had converged across the three models, ChatGPT showed the strongest GQS performance, and expert opinion scores favored ChatGPT and DeepSeek over Perplexity. Across all three models, Gunning Fog Index scores decreased in 2026, suggesting improved readability over time. Representative examples of year-on-year changes in chatbot responses to selected guideline-derived clinical questions are presented in Table 5.

**Table 5:** Representative examples of year-on-year changes in chatbot responses to selected EAU guideline-derived male infertility questions

Clinical question	ChatGPT	Perplexity	DeepSeek
How should testicular volume be assessed?	**2025:** Recommends a combination: clinical examination using orchidometer plus ultrasound for higher accuracy. Suggests ellipsoid formula (L × W × H × 0.52) for ultrasound measurements. Provides reference values and clinical tips.	**2025:** Prefers ultrasound as the first choice (Lambert formula: L × W × H × 0.71), with orchidometer as a secondary option in clinical practice. Offers detailed technical explanations and a comparative table.	**2025:** Highlights orchidometer as the “gold standard” and places ultrasound in a secondary role. Presents a more classical and concise approach.
	**2026:** Distinguishes between routine bedside assessment with a Prader orchidometer and ultrasound when precise measurement is required. Describes ultrasound as the most accurate method, provides commonly used formulas including L × W × H × 0.52 and L × W × H × 0.71, and adds practical documentation and measurement tips.	**2026:** Maintains ultrasound as the most accurate and reproducible method, using standardized formulas including L × W × H × 0.71. Describes orchidometer and caliper-based measurements as practical but less precise bedside alternatives, and emphasizes consistency in serial measurements.	**2026:** Repositions ultrasound as the gold standard for accurate measurement and Prader orchidometer as a routine bedside tool. Provides the L × W × H × 0.71 formula, notes the limitations of orchidometry, and offers more structured clinical guidance.
How should semen analyses be performed?	**2025:** Describes WHO 2021 criteria in detail, including sample collection, transport, processing, and parameters (volume, motility, morphology, etc.). Emphasizes repeat testing and additional assessments.	**2025:** Provides a very detailed and extensive approach based on WHO 2021 criteria, including pre-analytical, analytical, and post-analytical phases. Summarizes using tables.	**2025:** Explains in detail based on WHO 6th edition (2021), including step-by-step process, advanced test recommendations, and summarized algorithms.
	**2026:** Describes WHO 6th edition-based standardized semen analysis, including collection conditions, laboratory timing, core semen parameters, and repeat testing. Emphasizes that results should be interpreted in the clinical context of the couple rather than as a simple fertile/infertile test.	**2026:** Provides a practical stepwise protocol covering abstinence, sample collection, transport, core semen parameters, and repeat testing. Offers clinically relevant guidance but is less explicitly WHO-focused and more general in style than the 2025 response.	**2026:** Summarizes a standardized WHO 6th edition-based semen analysis protocol, including abstinence, collection, transport, laboratory parameters, and repeat testing. More concise than the 2025 response, with less emphasis on advanced tests and algorithms.
What is the essential approach for indeterminate testicular lesions in infertility?	**2025:** Suggests a stepwise approach: clinical evaluation, tumor markers, repeat ultrasound, specialist referral, surgical options, and onco-TESE. Presented like a decision algorithm.	**2025:** Provides a very detailed conservative approach; recommends observation for small lesions and biopsy or testis-sparing surgery for high-risk cases. Emphasizes a multidisciplinary team approach.	**2025:** Discusses tumor risk assessment, MRI/CEUS, markers, biopsy, or organ-preserving surgical options. Highlights fertility preservation considerations.
	**2026:** Emphasizes risk-stratified, fertility-preserving management rather than automatic orchiectomy. Recommends ultrasound characterization, tumor markers, fertility assessment and sperm cryopreservation, with surveillance or testis-sparing surgery plus frozen section for low-risk indeterminate lesions and oncologic surgery for suspicious cases.	**2026:** Emphasizes multidisciplinary, risk-adapted management with a conservative bias for small non-palpable lesions. Recommends active surveillance in low-risk cases and testis-sparing surgery or orchiectomy when suspicious features, growth, or higher malignancy risk are present.	**2026:** Emphasizes fertility-preserving, risk-stratified management with active surveillance for small stable lesions and testis-sparing surgery with frozen section when intervention is needed. Highlights sperm cryopreservation and avoidance of reflex radical orchiectomy.

## Discussion

In this repeated guideline-based evaluation, we investigated how the responses of three widely used large language model platforms to male infertility-related clinical questions changed between 2025 and 2026. The principal finding was that chatbot performance was dynamic and model-specific rather than uniformly progressive. ChatGPT demonstrated consistent improvement across reliability, overall quality, readability, and expert-rated clinical usefulness. DeepSeek showed the most pronounced year-on-year improvement from a lower baseline. In contrast, Perplexity, although still capable of generating broadly accurate and detailed responses, showed a relative decline in GQS and expert opinion scores. These findings suggest that the clinical value of LLMs in male infertility cannot be assumed to improve automatically over time and should be reassessed periodically against stable guideline-based standards.

A key methodological feature of this study is that the same 88 EAU guideline-derived questions were used in both assessment years. Because the relevant male infertility recommendations remained largely stable between the 2025 and 2026 EAU Sexual and Reproductive Health Guidelines, the observed differences are more likely to reflect changes in model behavior, retrieval strategy, source selection, and response-generation quality rather than changes in the underlying clinical reference standard. This approach is particularly relevant for LLM evaluation because chatbot systems are not static resources. Their outputs may change because of model upgrades, retrieval modifications, safety tuning, and changes in source access or ranking.

The improvement observed with ChatGPT is clinically important. In 2025, ChatGPT responses were generally accurate and clinically useful, but some answers showed partial shifts in emphasis, limited source transparency, or incomplete guideline framing. In 2026, after transition to ChatGPT-5.5, the responses more clearly reflected guideline-oriented reasoning and more frequently provided identifiable supporting sources. This was consistent with the quantitative results, in which ChatGPT improved across all four scored domains. Our findings are also compatible with recent male infertility-specific literature. Gokmen et al. evaluated ChatGPT-4.0 using 1073 male infertility-related queries based on AUA/ASRM guidance and reported improvement across true/false, multiple-choice, and open-ended questions over a 60-day period, with open-ended accuracy increasing from 78% to 86% and responses becoming more comprehensive and clinically relevant over time [12].

However, the improvement in ChatGPT should not be interpreted as complete reliability. Kaplan-Marans et al. showed that a guideline-trained model, UroGPT, markedly outperformed standard ChatGPT-4o when answering EAU male infertility guideline-based questions. UroGPT produced 94% complete and accurate responses compared with 38% for standard ChatGPT, while standard ChatGPT produced a higher rate of incorrect or misleading responses [13]. This is directly relevant to our findings: even when general-purpose LLMs improve, constraining or grounding models in expert-authored guideline content may provide a more reliable pathway for clinical use. At the same time, the same study emphasized that even the guideline-trained model was not yet appropriate for direct patient care and should remain a research or academic tool at its current stage [13].

Perplexity demonstrated a different pattern. In the 2025 assessment, it was the only evaluated platform that consistently provided references, which likely contributed to its high DISCERN, GQS, and expert opinion scores. In the 2026 reassessment using Perplexity Sonar, many responses remained broadly correct and detailed, but the source orientation and clinical focus appeared less consistent. This distinction is important because citation availability alone does not guarantee clinical reliability. A response may appear trustworthy because it includes references, but the value of those references depends on their authority, relevance, and correct integration into the answer. Aljamaan et al. developed a Reference Hallucination Score to evaluate medical AI chatbot citations and showed that reference hallucination varies substantially among chatbots, emphasizing the importance of verifying both output and citations [5].

This point is particularly relevant to our Perplexity findings. In 2025, Perplexity’s citation-enabled structure appeared to provide an advantage. By 2026, however, our expert assessment suggested that the presence of citations did not always translate into higher global quality or clinical usefulness. The decline in GQS and expert opinion scores may reflect a shift from guideline-focused clinical synthesis toward broader, sometimes more generic, search-grounded responses. Recent work on deep research agents similarly cautions that systems with retrieval and citation capabilities may produce comprehensive and well-referenced outputs, yet citation fidelity, claim-source alignment, evidence ranking, and retrieval transparency remain unresolved limitations [14]. Therefore, citation-enabled AI systems should be evaluated not only by whether they provide references, but also by whether those references are clinically appropriate, authoritative, and correctly interpreted.

The most pronounced improvement was observed with DeepSeek. In 2025, DeepSeek often provided acceptable answers to broad questions but was more vulnerable to hallucination, overgeneralization, or clinically imbalanced statements in more specific male infertility scenarios. One representative example involved testicular volume assessment. In 2025, DeepSeek described the Prader orchidometer as the “gold standard” for clinical practice while also acknowledging ultrasonography as the most accurate method, resulting in ambiguous clinical prioritization. In 2026, DeepSeek V4 more clearly distinguished ultrasonography as the most accurate and objective method and the Prader orchidometer as a practical bedside tool. This qualitative improvement was consistent with the marked increase in DISCERN and expert opinion scores. Representative screenshots of the 2025 and 2026 DeepSeek responses to this question are provided in Supplementary Figures S1 and S2.

The observed improvement in DeepSeek is also supported by recent clinical benchmarking literature. Sandmann et al. evaluated DeepSeek-V3 and DeepSeek-R1 in clinical decision-making tasks and found that DeepSeek models performed at a level comparable to, and in some cases better than, proprietary LLMs, including GPT-4o [15]. Nevertheless, the authors also emphasized that many cases did not achieve maximum scores and that inaccurate predictions could pose potential risks if outputs were used for immediate medical decisions without expert oversight [15]. This aligns closely with our interpretation: DeepSeek’s improvement is promising, particularly given the potential of open or lower-cost models, but it does not eliminate the need for domain-specific validation and clinician supervision. Potential patient-safety concerns were identified in seven DeepSeek responses from 2025. These responses contained inaccurate, incomplete, or insufficiently qualified information, although none included an explicit diagnostic or therapeutic recommendation likely to cause immediate or serious harm. Nevertheless, apparent sophistication should not be mistaken for clinical reliability. Even fluent, well-structured, and convincing responses may contain errors, omissions, or inappropriate clinical prioritization. LLM outputs should therefore be read critically and verified against authoritative sources, and the absence of explicit high-risk advice should not be interpreted as evidence that these platforms are suitable for unsupervised clinical use.

The relationship between DISCERN, GQS, and expert opinion deserves particular attention. DISCERN primarily captures reliability-related features such as clarity, balance, uncertainty acknowledgment, and source transparency. GQS and expert opinion, in contrast, better reflect clinical synthesis, completeness, practical usefulness, and appropriate emphasis. In 2026, DISCERN scores converged across the three models, suggesting that all platforms had become more capable of producing responses with basic reliability markers. However, GQS and expert opinion scores continued to differentiate model performance. This indicates that superficial reliability features, including citation presence or structured formatting, are not sufficient to determine clinical usefulness in male infertility. For andrological practice, the quality of guideline interpretation and the appropriateness of clinical prioritization are as important as whether references are present.

Readability also improved over time. All three models showed lower Gunning Fog Index scores in 2026, suggesting that their responses became easier to read. This is encouraging because male infertility counseling often involves complex and emotionally sensitive information. However, readability should not be interpreted in isolation. A simpler response may be more accessible but still clinically unsafe if it is incomplete or inaccurate. Conversely, a more complex response may be valuable for clinicians but less useful for patients. The ideal AI-generated response in male infertility should therefore combine readability, guideline consistency, appropriate source selection, and explicit acknowledgment of uncertainty.

Our findings should also be considered in the broader context of AI applications in male infertility. Recent reviews suggest that AI may support the clinical management of male infertility in areas such as semen analysis, hypogonadism, assisted reproductive technology, sperm selection, sperm retrieval prediction, and patient communication. Naik et al. emphasized that AI may help integrate large volumes of data into predictive models and support patient counseling, while cautioning that the quality of care must remain high [16]. Similarly, Qaderi et al. mapped AI applications in male infertility within IVF and reported use cases including sperm morphology, motility, non-obstructive azoospermia sperm retrieval prediction, IVF success prediction, and sperm DNA fragmentation assessment; they emphasized the need for multicenter validation, standardized methods, and attention to ethical concerns such as data privacy before broader clinical implementation [17].

These considerations reinforce the clinical interpretation of our study. LLMs may now be increasingly useful as adjunctive tools for male infertility education, clinician preparation, and patient-facing explanation. They can rapidly summarize guideline-based information, structure complex topics, and identify relevant references. Nevertheless, their outputs should not be treated as autonomous clinical recommendations. The risk of hallucination, irrelevant or weak source selection, overconfident phrasing, and incomplete clinical context remains. This risk is particularly important in male infertility, where inaccurate advice may affect diagnostic timing, patient anxiety, treatment expectations, fertility preservation decisions, and reproductive planning.

This study has several strengths, including the use of the same 88 EAU guideline-derived questions in 2025 and 2026, a multidimensional scoring approach, and independent evaluation by two academic urologists with strong inter-rater agreement. The inclusion of representative qualitative examples also helped illustrate clinically meaningful changes in model behavior. However, the study has limitations. The comparator set did not include a medical-specific or clinically grounded retrieval-augmented system; therefore, the findings should not be interpreted as a comparison between general-purpose platforms and specialized clinical AI tools. Only a single response was retained for each prompt–model–year combination, and repeated querying was not performed. Consequently, the variability of LLM outputs across repeated queries could not be assessed, and the findings represent sampled first-pass outputs obtained at specific time points rather than stable estimates of model performance or reproducibility. Furthermore, because the questions were directly derived from EAU guideline statements, their lexical and conceptual similarity to the guideline may have facilitated the generation of guideline-concordant responses and may limit generalizability to vague, colloquial, or poorly specified user questions.

The study was also limited to English-language responses in male infertility, and the findings may not be generalizable to other languages, specialties, or multi-turn interactions. User-facing platforms may undergo undisclosed backend changes; therefore, the results should be interpreted as a platform-level reassessment rather than a fixed-model comparison. Finally, although expert scoring was blinded, some subjectivity remains, and the study evaluated information quality rather than real-world clinical decisions or patient outcomes.

## Conclusions

In this repeated evaluation of freely accessible, general-purpose LLM platforms using standardized EAU guideline-derived questions, performance changed between 2025 and 2026 in a model-specific rather than uniformly progressive manner. ChatGPT showed consistent improvement across all four scored domains, while DeepSeek demonstrated substantial improvement from a lower baseline. In contrast, Perplexity showed improved readability but lower global quality and expert-rated clinical usefulness in 2026, indicating that citation availability alone does not ensure guideline-concordant or clinically useful information. Potential patient-safety concerns were identified in seven DeepSeek responses from 2025, although none of the 528 responses contained an explicit recommendation considered likely to cause immediate or serious patient harm.

These findings should be interpreted as sampled first-pass outputs obtained from general-purpose platforms at specific time points, rather than stable estimates of model performance or response reproducibility. The use of clearly formulated questions retaining terminology from the EAU guidelines may also have facilitated the generation of guideline-concordant responses and may limit generalizability to less specific user queries. Moreover, the study did not compare these platforms with medical-specific or clinically grounded retrieval-augmented systems. LLMs may support rapid access to and summarization of male infertility information, but fluent and well-structured responses should not be assumed to be clinically accurate or safe. Verification against authoritative sources and appropriate human oversight remain necessary.

## Electronic supplementary material

Below is the link to the electronic supplementary material.

Supplementary Material 1

Supplementary Material 2

Supplementary Material 3

## Data Availability

All data generated or analyzed during this study are included in this article and its supplementary information files. Additional data may be available from the corresponding author on reasonable request.

## Abbreviations

AI: Artificial intelligence
AUA: American Urological Association
DISCERN: DISCERN instrument for reliability of health information
EAU: European Association of Urology
GFI: Gunning Fog Index
GQS: Global Quality Score
ICSI: Intracytoplasmic sperm injection
IVF: In vitro fertilization
LLM: Large language model
SPSS: Statistical Package for the Social Sciences
TRIPOD-LLM: Transparent Reporting of a multivariable prediction model for Individual Prognosis or Diagnosis—Large Language Models
