# Supplementary material for: Evolution of guideline-based male infertility responses by large language models between 2025 and 2026: a comparative analysis of ChatGPT, Perplexity, and DeepSeek
Source: Basic Clin Androl. 2026 Oct 16;36:30. doi: 10.1186/s12610-026-00327-z (PMC13675980; doi:10.1186/s12610-026-00327-z)
Supplement: Supplementary file 1 [file 12610_2026_327_MOESM3_ESM.docx]

**Supplementary Figure S1. DeepSeek response to the clinical question “How should testicular volume be assessed?” in 2025.**
In the 2025 response, DeepSeek described the Prader orchidometer as the clinical “gold standard” and placed ultrasonography in a secondary role, reflecting an imbalanced clinical framing of testicular volume assessment.

**Supplementary Figure S2. DeepSeek response to the clinical question “How should testicular volume be assessed?” in 2026.**
In the 2026 response, DeepSeek provided a more structured comparison of assessment methods and described ultrasonography as the gold standard for accurate, reproducible, and objective measurement, while positioning the Prader orchidometer as a practical bedside tool.
